# The effect of short-term refrigeration on platelet responsiveness

**DOI:** 10.1038/s41598-022-21124-4

**Published:** 2022-10-07

**Authors:** Anna Kobsar, Karina Koehnlechner, Philipp Klingler, Marius Niklaus, Julia Zeller-Hahn, Angela Koessler, Katja Weber, Markus Boeck, Juergen Koessler

**Affiliations:** Institute of Clinical Transfusion Medicine and Haemotherapy, University of Wuerzburg, Oberduerrbacher Straße 6, 97080 Wuerzburg, Germany

**Keywords:** Cell adhesion, Cell signalling, Senescence, Surgery, Drug regulation, Haematological cancer, Drug development

## Abstract

Storage of platelet concentrates (PC) at cold temperature (CT) is discussed as an alternative to the current standard of storage at room temperature (RT). Recently, we could show that cold-induced attenuation of inhibitory signaling is an important mechanism promoting platelet reactivity. For developing strategies in blood banking, it is required to elucidate the time-dependent onset of facilitated platelet activation. Thus, freshly prepared platelet-rich-plasma (PRP) was stored for 1 and 2 h at CT (2–6 °C) or at RT (20–24 °C), followed by subsequent comparative analysis. Compared to RT, basal and induced vasodilator-stimulated phosphoprotein (VASP) phosphorylation levels were decreased under CT within 1 h by approximately 20%, determined by Western blot analysis and flow cytometry. Concomitantly, ADP- and collagen-induced threshold aggregation values were enhanced by up to 30–40%. Furthermore, platelet-covered areas on collagen-coated slides and aggregate formation under flow conditions were increased after storage at CT, in addition to induced activation markers. In conclusion, a time period of 1–2 h for refrigeration is sufficient to induce an attenuation of inhibitory signaling, accompanied with an enhancement of platelet responsiveness. Short-term refrigeration may be considered as a rational approach to obtain PC with higher functional reactivity for the treatment of hemorrhage.

In transfusion medicine, platelet concentrates (PC) are frequently manufactured blood components, particularly used for prophylaxis or treatment of hemorrhage due to thrombocytopenia or for patients with reduced platelet function^1^. Currently, PC are regularly stored at room temperature (RT, 20–24 °C), accompanied by two major disadvantages: the risk of bacterial growth and the development of functional restrictions called storage lesions^2,3^. Therefore, according to manufacturing conditions and legal regulations, the shelf life of PC is commonly restricted to 4–7 days in many countries. Platelet storage at cold temperature (CT, 2–6 °C), which was performed until the 1980s, offers an alternative to reduce the risk of bacterial growth and to prolong the storage period^4–6^. RT has become the standard for platelet storage, since refrigeration induces clustering of glycoprotein Ib (GPIb) on the platelet surface and desialylation resulting in rapid clearance of re-transfused platelets in vivo^7,8^, although cold-stored platelets are considered to be superior in acute hemorrhage due to increased responsiveness^2^.

Recently, we could demonstrate that higher platelet reactivity upon cold storage is mediated by attenuation of inhibitory signaling, contributing to enhanced ADP-induced aggregation responses^9^. Vasodilator-stimulated phosphoprotein (VASP) phosphorylation, as a representative marker of platelet inhibition^10^, developed continuously decreasing basal and induced levels during storage at CT for five days.

The continuous provision of two different PC specifications in parallel—RT-stored PC for patients with chronic thrombocytopenia and RT-stored PC for patients with acute hemorrhage—would be an organizational challenge. However, after initial storage at RT, it may be an opportunity to expose platelets at CT for a short time period, with the aim to instantly obtain platelets with higher hemostatic capacity “on demand”. In this respect, it would be mandatory to explore the time-dependent course of molecular and functional changes during early platelet refrigeration.

Therefore, in this study, we analyzed the effects on platelet responsiveness induced by short-term refrigeration for 2 h. For experimentation, we used platelet-rich-plasma, as a material very similar to PC, to avoid the waste of valuable PC required for patient care. The analytical procedures comprised inhibitory signaling, activation markers, chemokine release, aggregation and adhesion or aggregate/thrombus formation under flow conditions, as important characteristics of platelet integrity.

## Results

### Cold storage did not relevantly affect platelet count in PRP

The platelet count in PRP did not significantly change under cold storage with values of 350 ± 71 × 10^3^/µL after 1 h and 337 ± 121 × 10^3^/µL after 2 h, compared with 389 ± 77 × 10^3^/µL at RT storage (n = 7, values as mean ± standard deviation).

### Basal and induced VASP phosphorylation decreases within 1 h of refrigeration

**Figure 1:**
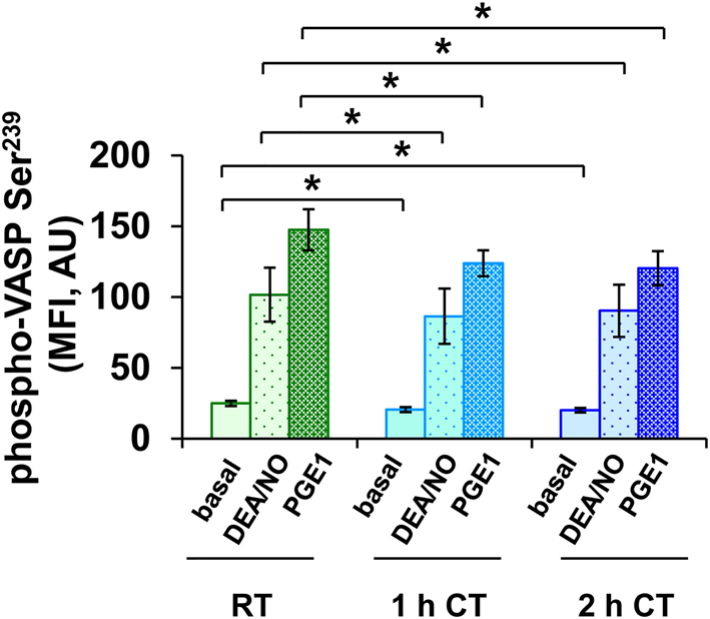
VASP phosphorylation is diminished after short-term refrigeration. PRP samples, stored at RT, at CT for 1 h or at CT for 2 h, were stimulated with 1 µM DEA/NO, 1 µM PGE1 or with buffer. After fixation and permeabilization, platelets were stained with an FITC-conjugated phospho-VASP-Ser^239^ antibody. The samples were analyzed by flow cytometry using a FACS Calibur flow cytometer (Becton Dickinson, Franklin Lakes, NJ, USA). VASP phosphorylation levels were measured as mean fluorescence intensity (MFI) in arbitrary units (AU). The histograms show the MFI values as mean AU ± SEM; n = 7; *: *p* < 0.05, as indicated.

VASP phosphorylation at Ser^239^, measured by flow cytometry, showed a decreasing tendency with basal values of 20.8 ± 1.8 AU (arbitrary units) at 1 h and 20.0 ± 1.6 AU at 2 h of cold storage, compared to 25.3 ± 1.9 AU at RT (Fig. 1). NO-induced levels were 96.6 ± 19.5 AU at RT and 80.2 ± 19.8 AU at 1 h or 84.7 ± 18.5 AU at 2 h of refrigeration. PGE1-induced levels were 147.5 ± 15.6 AU at RT, 124.0 ± 9.9 AU at 1 h or 120.7 ± 12.9 AU at 2 h.

**Figure 2:**
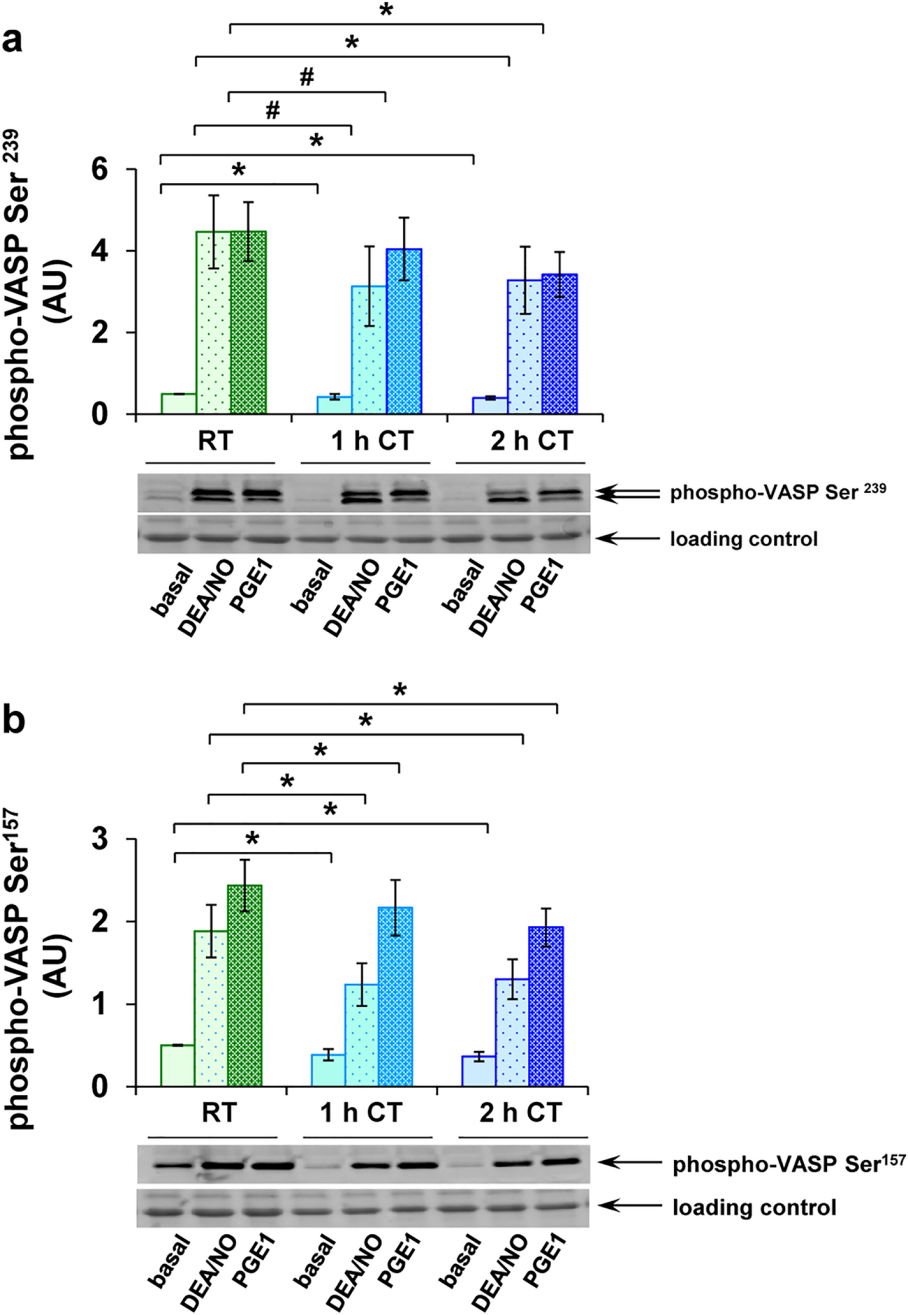
Western blot analysis of VASP phosphorylation confirms attenuated inhibitory signaling after short-term refrigeration. PRP samples, stored at RT, at CT for 1 h or at CT for 2 h, were stimulated with 1 µM DEA/NO, 1 µM PGE1 or with buffer. Consecutively, WP were prepared, lysed and analyzed by Western blot using phospho-VASP Ser^239^ (**a**) and phospho-VASP Ser^157^ (**b**) antibodies. The histograms show the levels of phosphorylated VASP relative to loading controls in arbitrary units (AU) as mean ± SEM; n = 7; *: *p* < 0.05, #: *p* < 0.1, as indicated. The original blots are presented in the supplementary Fig. S1 online.

These results were confirmed in Western blot analysis, including the detection of VASP phosphorylation of both Ser^239^ and Ser^157^ (Fig. 2). Levels at Ser^239^ of 0.50 ± 0.01 AU dropped to 0.38 ± 0.05 AU after 1 h and to 0.41 ± 0.04 AU after 2 h of refrigeration (Fig. 2a). DEA/NO-induced levels were decreased to 2.99 ± 1.04 AU after 1 h and to 3.07 ± 0.85 AU after 2 h at CT, compared to 4.12 ± 0.86 AU at RT. PGE-induced levels with 4.21 ± 0.71 AU at RT were reduced during cold storage to 3.84 ± 0.79 AU and 3.42 ± 0.55 AU after 1 h and 2 h of refrigeration, respectively.

A similar pattern was observed for VASP phosphorylation at Ser^157^, with values of 0.50 ± 0.01 AU at RT, 0.38 ± 0.08 AU after 1 h and 0.34 ± 0.06 AU after 2 h at CT (Fig. 2b). DEA/NO-induced values developed reduced values from 1.93 ± 0.34 AU under RT to 1.25 ± 0.29 AU at 1 h and 1.33 ± 0.27 AU at 2 h under CT. PGE1-induced values of 2.46 ± 0.32 AU at RT were diminished to 2.20 ± 0.36 AU and to 1.82 ± 0.24 AU after 1 h or 2 h at CT, respectively.

### Threshold aggregation is supported within 1 h of refrigeration

**Figure 3:**
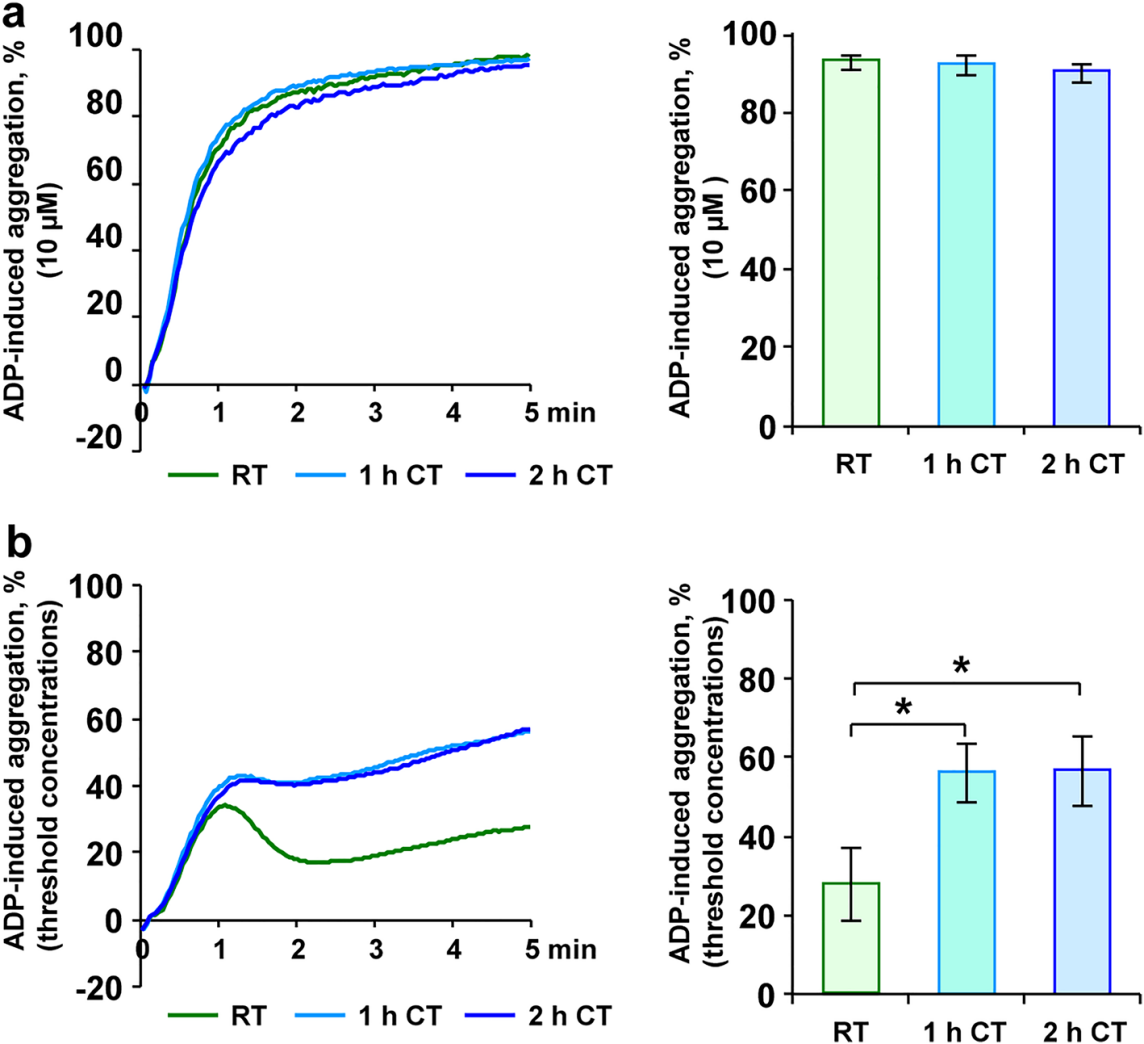
ADP-induced threshold aggregation is enhanced upon short-term refrigeration. PRP samples, stored at RT, at CT for 1 h or at CT for 2 h, were stimulated with 10 µM ADP (**a**) or with ADP in individual threshold concentrations (0.5–3.0 µM) (**b**). After that, light transmission aggregometry was continuously measured for 5 min. Mean aggregations traces are shown (left) and maximal aggregation values are presented as mean ± SEM (right); n = 6; *p < 0.05, as indicated.

**Figure 4:**
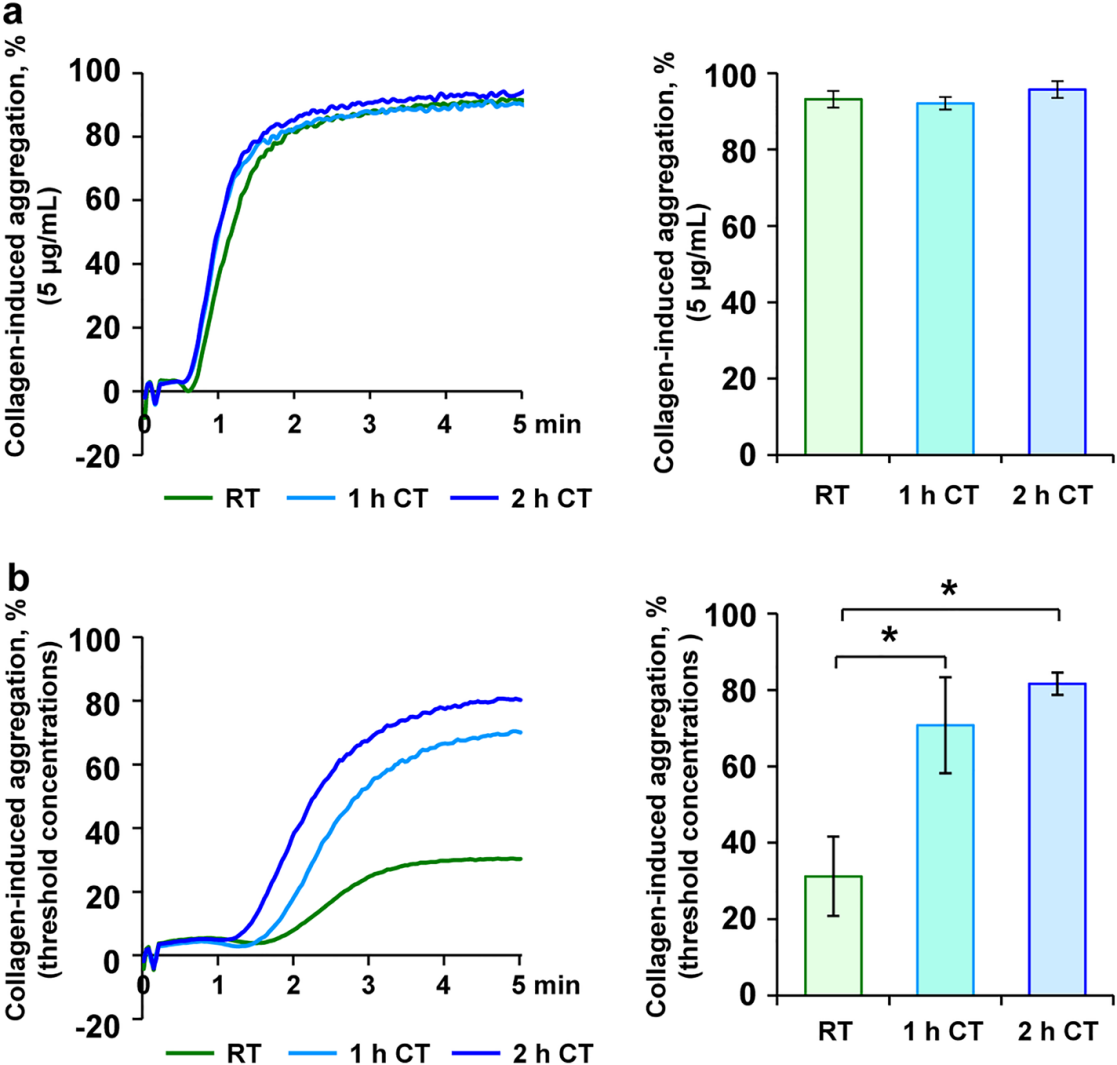
Collagen-induced threshold aggregation is enhanced upon short-term refrigeration. PRP samples, stored at RT, at CT for 1 h or at CT for 2 h, were stimulated with 10 µg/mL collagen (**a**) or with collagen in threshold concentrations (0.5–1.0 µg/mL) (**b**). After that, light transmission aggregometry was continuously measured for 5 min. Mean aggregations traces are shown (left) and maximal aggregation values are presented as mean ± SEM (right); n = 5; *p < 0.05, as indicated.

Irreversibly induced aggregation using 10 µM ADP or 10 µg/mL collagen was not affected by CT, reaching more than 90% (Fig. 3a and Fig. 4a). Threshold ADP-induced aggregation of 27.7 ± 7.8% is supported after 1 h at CT with 56.4 ± 6.3% and similarly after 2 h with 57.0 ± 7.4% (Fig. 3b). The same effect was observed for the agonist collagen in threshold concentrations, inducing 31.2 ± 10.4% at RT and 70.8 ± 12.6% after 1 h at CT or 81.7 ± 3.0% after 2 h at CT (Fig. 4b).

### Adhesion and aggregate formation are increased under refrigeration

**Figure 5:**
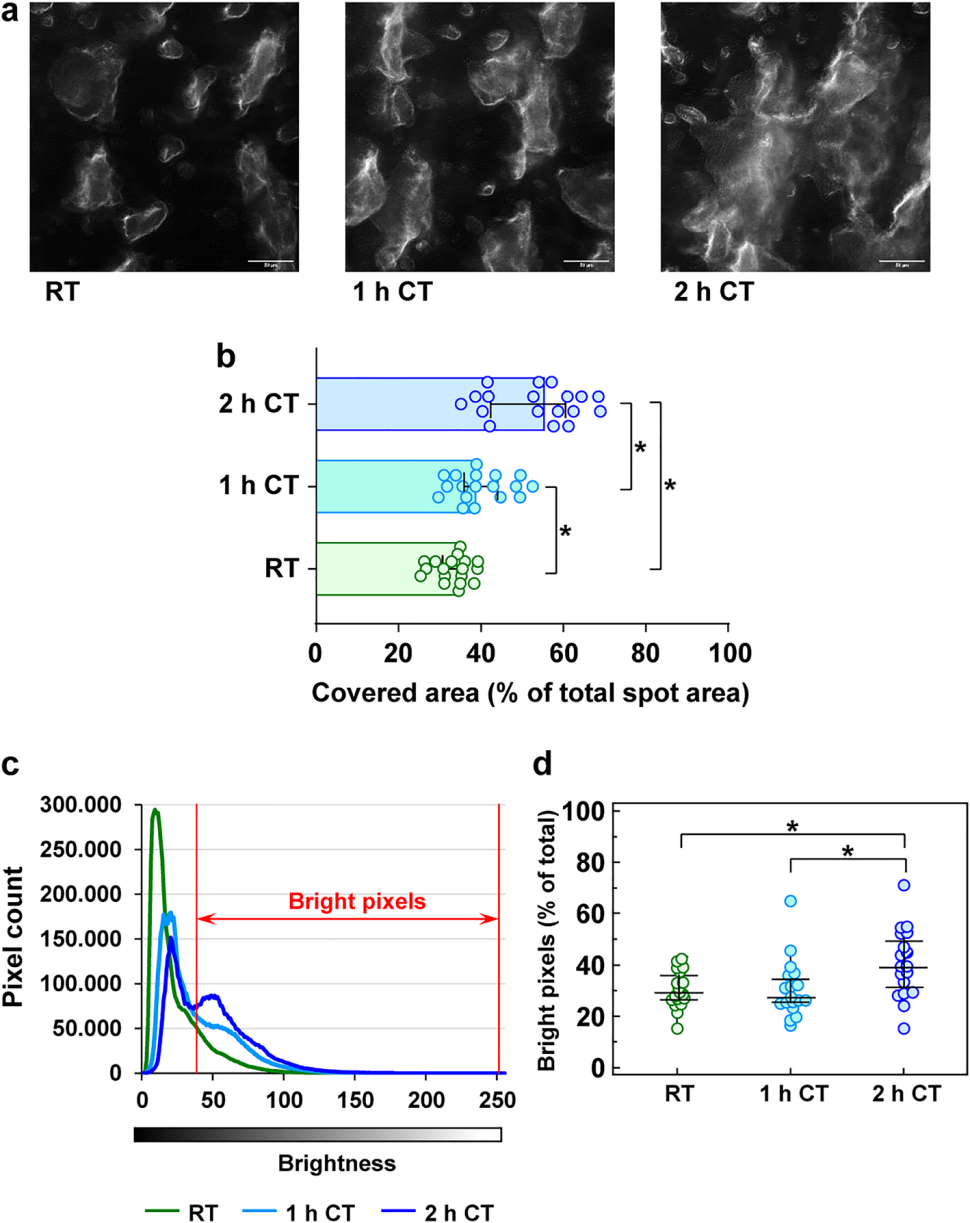
Short-term refrigeration supports platelet adhesion and aggregate formation. Diluted PRP, stored at RT, at CT for 1 h or at CT for 2 h, was perfused through a collagen-coated flow chamber by means of a syringe pump. The formation of platelet aggregates on the surface of slides was visualized by high-resolution, phase-contrast bright-field imaging using a live cell imaging system with a Nikon Eclipse Ti2 microscope (Nikon GmbH, Duesseldorf, Germany) and a CFI Plan Fluor DLL 60 × oil immersion objective. Representative images of covered slides are shown (**a**) and the percentages of covered area for the different PRP samples are given as median ± IQR (interquartile range from quartile 1 to quartile 3) (**b**). The density and the coating thickness of covering platelet layers were determined by analysis of pixel brightness (grey scale 0–256, 8 bit) at settled spots (**c**). The percentage of bright pixels (with brightness > 37.5) for the different PRP samples (**d**) are presented as median ± IQR; n = 3; *p < 0.05, as indicated.

Under flow conditions on collagen-coated slides (Fig. 5a), platelet adhesion is enhanced with a median covered area of 38.9% (interquartile range, IQR, from 35.8% to 44.0%) after 1 h and 55.0% (IQR from 41.8% to 61.0%) after 2 h of cold storage, in comparison to RT-storage with a median covered area of 34.3% (IQR from 30.9% to 35.2%) (Fig. 5b). In addition, ongoing refrigeration enhanced the coating thickness of aggregates, indicated by higher brightness of covered area (Fig. 5c,d). The fraction of bright pixels rose from median 29.2% (IQR from 26.9% to 35.4%) at RT and 27.1% (IQR from 25.4% to 34.3%) at CT for 1 h up to 39.3% (IQR from 31.1% to 49.4%) at CT for 2 h.

### Refrigeration supports pre-activation of platelets and chemokine release only to a minor degree

**Figure 6:**
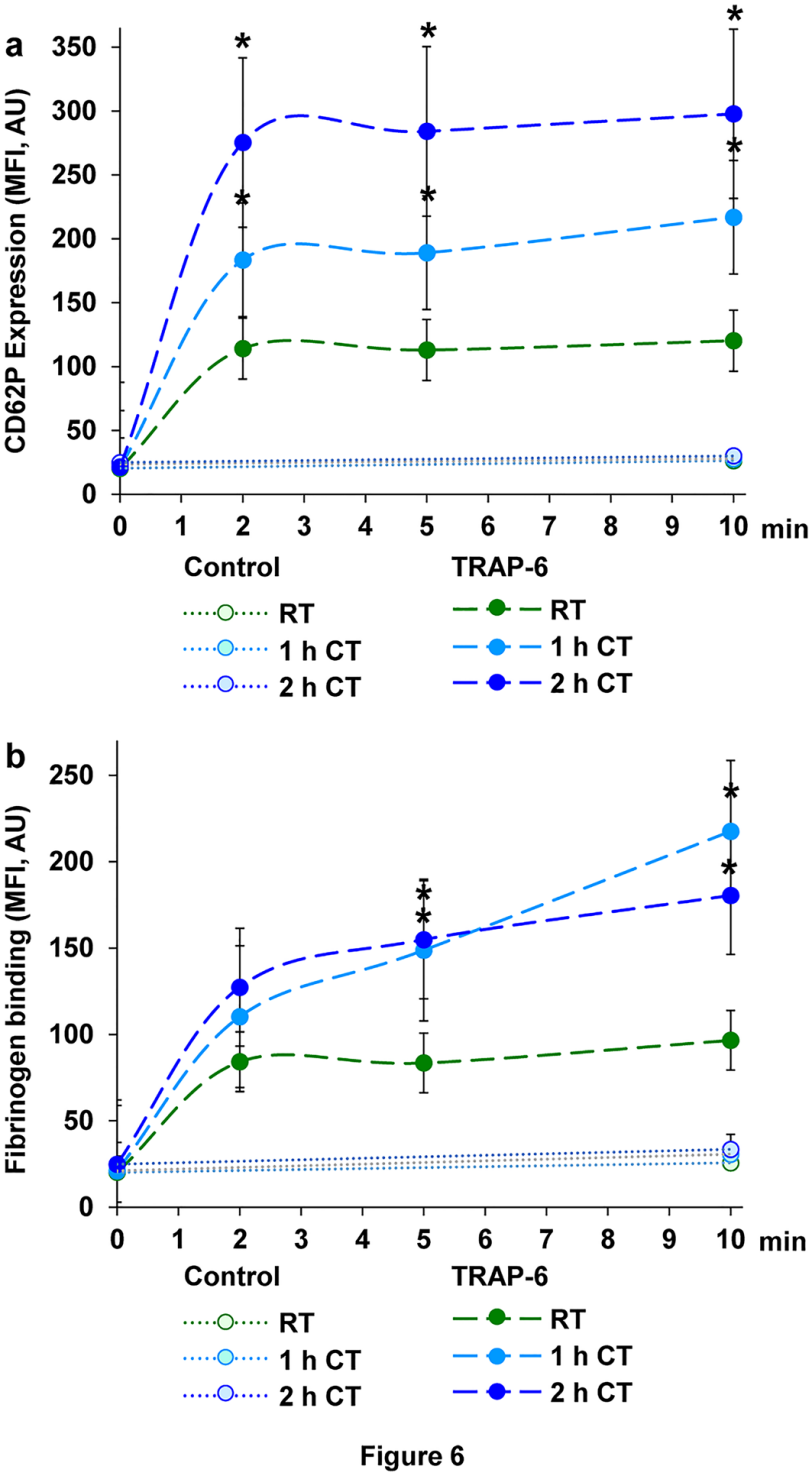
Short-term refrigeration induces slight pre-activation of platelets. CD62P expression and fibrinogen binding as activation markers were measured by flow cytometry. PRP samples, stored at RT, at CT for 1 h or at CT for 2 h, were stimulated with 10 µM TRAP-6 for 2–10 min and stained with a FITC-conjugated anti-CD62P antibody (**a**) or a FITC-conjugated anti-fibrinogen antibody (**b**). Control samples represent unstimulated platelets stained with FITC-conjugated anti-CD62P or anti-fibrinogen antibody. The analysis was performed with a FACS Calibur flow cytometer (Becton Dickinson, Franklin Lakes, NJ, USA) by detection of mean fluorescence intensity (MFI). The diagrams show the MFI values as mean ± SEM; n = 6; *p < 0.05, as indicated.

Basal CD62P expression and basal fibrinogen binding, as essential markers of platelet activation, were not relevantly affected by cold storage for 1 h or 2 h (Fig. 6a,b). However, CD62P expression stimulated by TRAP-6 for 2 min was pronounced in refrigerated samples with 189.0 ± 33.7 MFI at 1 h of CT and 284.0 ± 59.5 MFI at 2 h of CT compared with 112.9 ± 7.8 MFI at RT (Fig. 6a). These effects also remained stable after extended TRAP-6 stimulation for 10 min. Similarly, but reaching peak values after 10 min of stimulation, TRAP-6-induced fibrinogen with 96.6 ± 12.6 MFI at RT was elevated to 217.6 ± 44.2 MFI at CT for 1 h and to 180.4 ± 23.3 MFI at CT for 2 h (Fig. 6b).

**Figure 7:**
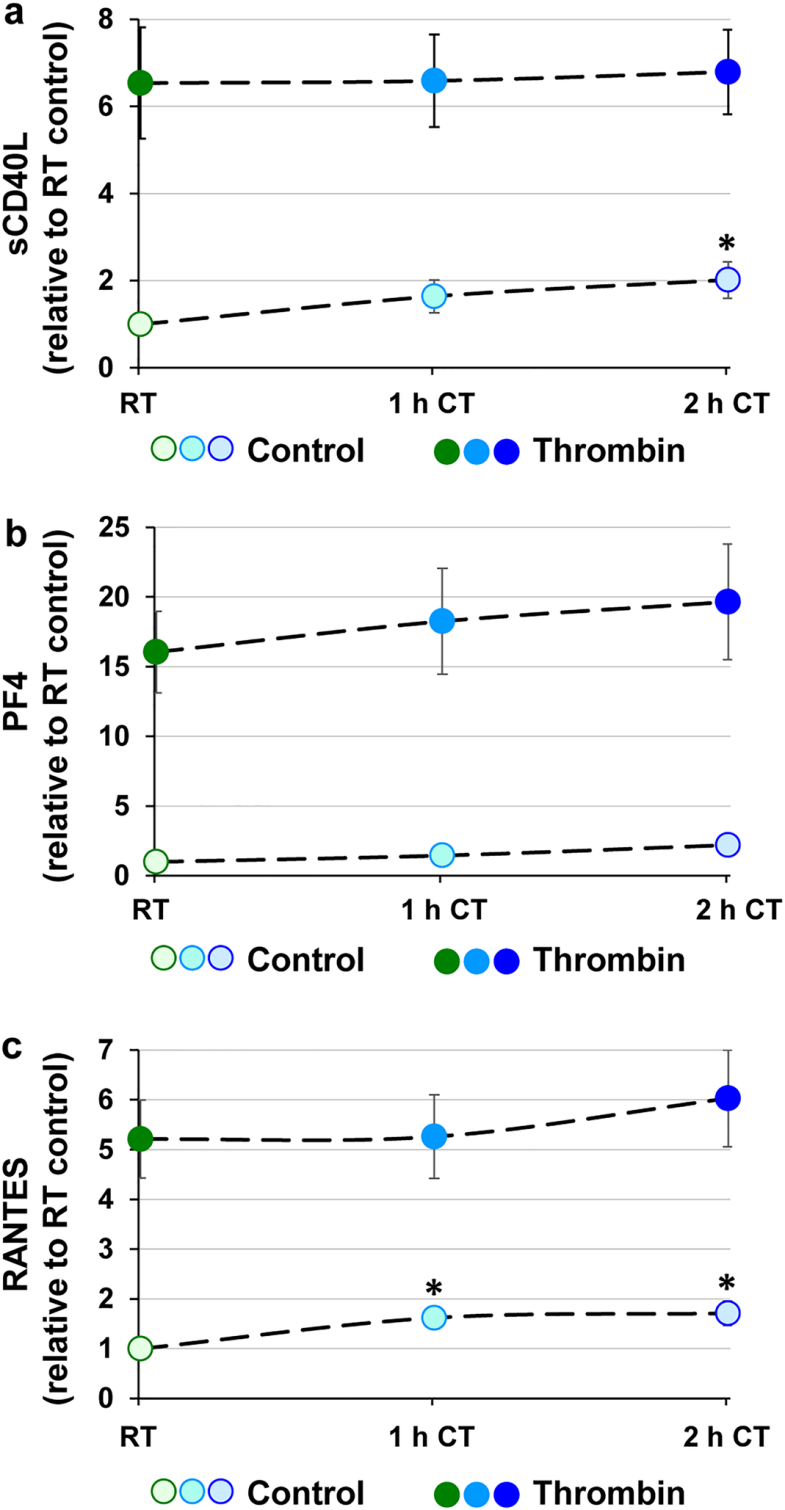
Chemokine release is slightly increased upon short-term refrigeration. PRP samples, stored at RT, at CT for 1 h or at CT for 2 h, were unstimulated (treated with buffer only) or stimulated with 0.5 U/mL thrombin for 30 min. After that, released levels of sCD40L (**a**), PF4 (**b**) and RANTES (**c**) were determined in the supernatants by immunoassay kits. The levels are calculated as fold stimulation relative to RT samples. The results are presented as mean ± SEM; n = 8; *p < 0.05, compared with RT.

Platelets contain a high number of chemokines, among them CD40L (soluble CD40 ligand), PF4 (platelet factor 4) or RANTES (Regulated on activation, normal T cell expressed and secreted), which are released upon platelet activation. Compared with RT, these chemokines showed slightly increasing levels in the supernatant of unstimulated platelets under CT (Fig. 7). In average, the levels of sCD40L were 1.6 ± 0.4 fold higher after 1 h and significantly 2.0 ± 0.4 fold higher after 2 h (Fig. 7a). PF4 levels had a tendency towards higher levels at CT with 1.5 ± 0.3 fold after 1 h (p = 0.09) and 2.2 ± 0.7 fold after 2 h (p = 0.06) (Fig. 7b). RANTES levels were elevated 1.6 ± 0.2 fold after 1 h and 1.7 ± 0.3 fold after 2 h of refrigeration (Fig. 7c).

Thrombin stimulation produced increments of 6.5 ± 1.3 fold (sCD40L), 16.0 ± 2.9 fold (PF4) or 5.2 ± 0.8 -fold (RANTES) under RT, much larger, but not different between RT- and CT-stored platelets (Fig. 7a–c).

## Discussion

Refrigeration of platelets is discussed as an alternative to the current standard of RT storage, providing two major advantages: the lower risk of bacterial growth in platelet-containing blood components and the elevation of platelet responsiveness for improved hemostatic effects in the treatment of hemorrhage^5^. Here, we address the time-dependent onset of cold-induced effects contributing to increased platelet reactivity.

The attenuation of inhibitory signaling has been identified as a progressive cold-induced mechanism supporting platelet responses during ex vivo storage of platelets for several days^9^. In this context, VASP is one of the major common substrates for both protein kinase A or protein kinase G and its phosphorylation is a sign of platelet inhibition^10,11^, keeping GPIIb/IIIa in the resting conformation, and in consequence, supporting the inhibition of fibrinogen binding, adhesion, and aggregation^12,13^.

In this study, basal VASP phosphorylation was decreased by approximately 20% after 1 and 2 h of cold storage in comparison to RT. Concomitantly, DEA/NO- or PGE1-induced phosphorylation peak levels were reduced by 10–20%, resulting in decreased increments. The suppressing effect on VASP phosphorylation was consistently detectable with both methods, flow cytometry and Western Blot analysis, and reliably detectable after 1 h of cold storage, as the earliest time-point of analysis.

In a recent study focusing at cold-stored PC for 7 days^9^, the increment of VASP phosphorylation at Ser^239^, induced by DEA/NO or PGE1, was decreased from 4–fivefold at RT to approximately 1.5–1.8 fold at CT after 2 days of storage. A similar effect was observed for the phosphorylation site Ser^157^.

According to the results, it appears that the attenuation of inhibitory signaling is rapidly initiated within 1 h of refrigeration, but does not reach its maximum compared with long-term refrigeration. However, this short period of refrigeration and this partial impairment of inhibitory signaling were sufficient to induce a reliably measurable increase of platelet responsiveness.

The reduction of VASP phosphorylation was accompanied with facilitated aggregation responses, as the major functional feature of platelets. In freshly prepared PRP, platelet aggregation is maintained for several hours and not yet affected by storage lesion as observed on day 1 or later^14,15^. For both used activators—ADP and collagen in threshold concentrations—an enhancement of induced aggregation up to 30–40% was detectable, pointing to the onset of promoted reactivity within 1 h of storage at CT. These findings were also confirmed by adhesion studies and analysis of aggregate formation under flow conditions, illustrated by larger covered areas and stronger aggregate formation with short-term refrigerated platelets on collagen-coated surfaces.

Unlike VASP phosphorylation or aggregation responses, these effects were increasing from time-points 1 h to 2 h of cold storage. Apart from different sensitivities of methods, this may be caused by cold-induced binding of von Willebrand factor to glycoprotein Ib on the platelet surface contributing to aggregate formation^16^.

Augmented platelet reactivity upon exposition to CT is also characterized by elevated activation markers^2,17^. In long-term refrigerated platelets, the levels were 3–fourfold higher for basal CD62P expression or basal fibrinogen binding after 2 days of cold storage, further progressing to 4–fivefold levels on day 5^9^. After 1 or 2 h at CT, basal values of CD62P expression and fibrinogen binding were unchanged, but TRAP-6-induced levels were 2–threefold higher in platelets at CT than in platelets at RT, indicating supported platelet reactivity in cold-stored platelets.

In addition, short-term refrigeration only led to minor spontaneous release of chemokines like RANTES, PF4 or sCD40L, as biological response modifiers with the potential to induce adverse transfusion-associated events or aggregation formation in products^18,19^. Thrombin-induced levels of released chemokines were comparable after different storage temperatures.

The major disadvantage of cold storage is the clustering of GPIb on the platelet surface resulting in accelerated clearance from the circulation after re-transfusion by hepatic macrophages^20^. This phenomenon may occur within 2 h of cold exposition, as observed in mice transfused with cold-stored platelets^7^. Otherwise, former “temperature cycling” experiments, with interruption of long-term cold storage by re-warming phases at 37 °C for 30 min, showed reversible cold-induced morphological lesions and elevated life spans of platelets in comparison to continuous cold storage^21,22^.

Therefore, for the amendment of storage strategies, it will be an important issue for further studies to thoroughly investigate the onset and reversibility of effects mediated by short-term cold storage. In cases of cold-stored PC not used for acute hemorrhage, it is mandatory to know, how re-storage at RT will influence clearing mechanisms and the development of platelet function.

As a limitation, it should be kept in mind that investigations were performed in vitro. Clinical studies are required to confirm improved hemostatic effects by short-term refrigeration, as recently demonstrated for long-term refrigerated PC contributing to reduced postoperative blood loss in cardiothoracic surgery^23^ or for the shortening of bleeding times^24,25^.

Furthermore, it should be mentioned that results refer to the milieu of PRP, prepared from freshly donated blood samples. It is mandatory to additionally analyze platelets from PC, which are stored for different time periods under blood banking standards with regulatory specifications and the use of appropriate storage materials. However, fresh platelet samples, as PRP from peripheral blood or as PRP from PC, are functionally comparable^9^ and the waste of PC has been avoided in this way. Regarding future studies, the novel data obtained in this work will be a substantial basis for comparison.

**Figure 8:**
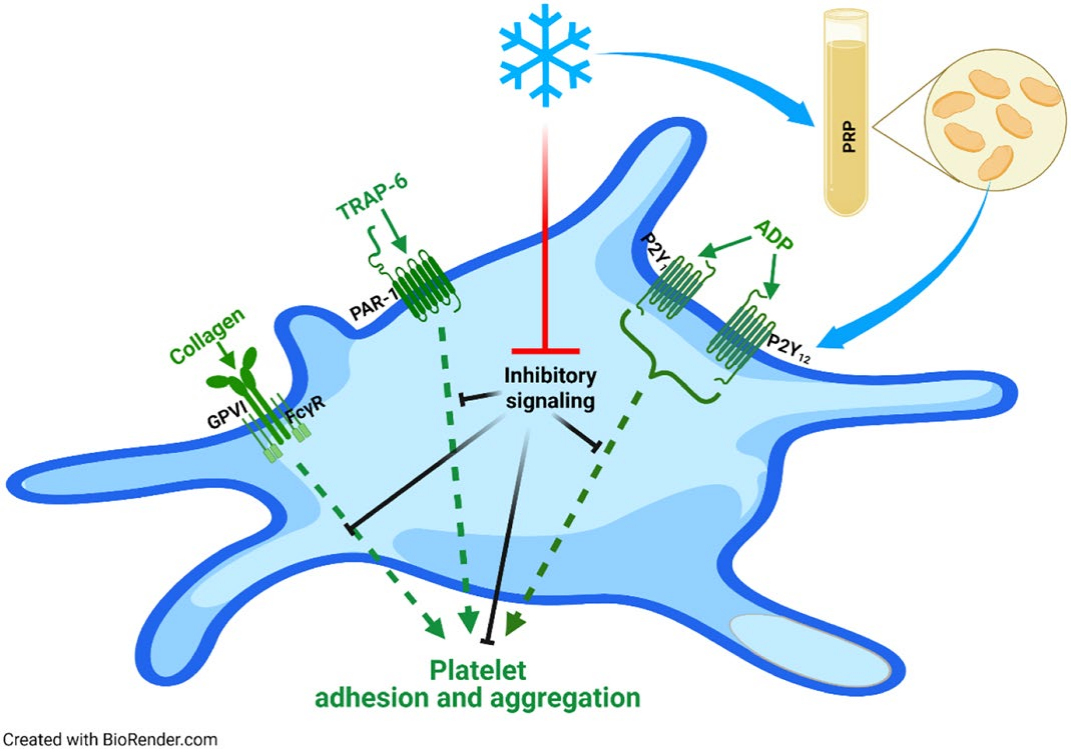
Mechanism of enhanced platelet reactivity by refrigeration. Attenuated inhibitory signaling facilitates ADP- and collagen-induced aggregation and platelet adhesion or aggregate formation. The figure was created with the software BioRender (URL: https://biorender.com, version used on 2022-06-30).

In summary, cold-induced attenuation of inhibitory signaling begins within 1–2 h of refrigeration, associated with an increase of platelet responsiveness as expressed by enhanced aggregation, adhesion and aggregate formation on coated surfaces, and induced expression of activation markers (Fig. 8). Short-term refrigeration may represent a practical approach to rapidly obtain PC with higher hemostatic capacity with time-points “1 h” or “2 h” as rational suggestions for the design of consecutive studies. The additional investigation of earlier time-points would also be of interest, since it is desirable to provide platelets with higher reactivity within less than 1 h in emergency situations.

## Materials and methods

### Materials

ADP was obtained from Haemochrom Diagnostica GmbH (Essen, Germany), collagen reagent HORM was from Takeda (Linz, Austria), thrombin receptor-activating peptide-6 (TRAP-6) from BACHEM (Weil am Rhein, Germany). Mouse monoclonal FITC-conjugated anti-fibrinogen antibody and the corresponding isotype were from STAGO Germany (Düsseldorf, Germany), FITC-conjugated mouse anti-CD62P antibody and the corresponding isotype control were from OriGene Biomedical GmbH (Burladingen, Germany). Prostaglandin E1 (PGE1), 4-[2-hydroxyethyl]-1-piperazineethanesulfonic acid (HEPES), Ponceau S, Tyrode’s salt solution, Ethylene glycol-bis(β-aminoethyl ether)-N,N,N',N'-tetraacetic acid (EGTA), bovine serum albumin (BSA), human plasma fibrinogen and FITC-conjugated goat anti-mouse antibody were from Sigma-Aldrich Chemie GmbH (Muenchen, Germany). Nitric oxide (NO) donor DEA/NONOate (DEA/NO) was from Enzo Life Sciences GmbH (Loerrach, Germany). Hank's Balanced Salt Solution (HBSS) was from Thermo Fisher Scientific GmbH (Dreieich, Germany). Mouse monoclonal phospho-VASP Ser^239^ and phospho-VASP Ser^157^ antibodies were from Nanotools (Teningen, Germany). StarBright Blue 700 conjugated goat anti-rabbit and anti-mouse antibodies were from Bio-Rad Laboratories, Inc. (Muenchen, Germany). RANTES, PF4 and sCD40L ELISA Kits were from R&D Systems GmbH (Wiesbaden-Nordenstadt, Germany).

### Blood collection and preparation of platelet-rich-plasma

Our studies with human platelets and the consent procedure were approved by our local ethics committee of the University of Wuerzburg (approval number 101/15). The participants provided their written informed consent to participate in this study. The study was performed according to our institutional guidelines and to the Declaration of Helsinki.

Venous whole blood samples were obtained from informed healthy voluntary donors without any medication intake. Peripheral blood was collected in polypropylene tubes containing 3.2% citrate buffer (106 mM trisodium citrate, Sarstedt, Nuembrecht, Germany).

Platelet-rich-plasma (PRP) was prepared by centrifugation of whole blood at 280 × *g* for 5 min (min) as described^10^.

Blood cell count was performed with the hematology analyzer Sysmex KX-21 N (Sysmex Europe GmbH, Norderstedt, Germany). PRP was divided into three parts (each 4.5 mL PRP in a polypropylene tube), which were stored at RT (20–24 °C) for 2 h (control), stored for 1 h at CT (2–6 °C) or stored for 2 h at CT. Before consecutive analysis, platelets were left quiescent at RT for 30 min.

### Flow cytometric detection of platelet VASP phosphorylation

For detection of VASP phosphorylation, similar to^26^, 30 µL PRP was stimulated with buffer, 1 µM DEA/NO or 1 µM PGE1 (final concentration) for 5 min at 37 °C, followed by 10 min fixation with 2.5% formaldehyde at RT. Consecutively, the samples were centrifuged for 1 min at 20,000 × *g*, and the pellets were permeabilized in 50 µL of PBS/BSA/Glc buffer (Dulbecco’s PBS (Ca^2+^, Mg^2+^ free), 5.5 mM D-glucose, 0.5% BSA) containing 0.2% Triton X-100 for 10 min at RT. Permeabilized platelets were stained for 30 min at RT with 0.5 µg of FITC-conjugated phospho-VASP-Ser^239^ antibody in the dark. Finally, all samples were diluted with 500 µL of PBS/BSA/Glc buffer and analyzed by flow cytometry using a FACS Calibur flow cytometer from Becton Dickinson (Franklin Lakes, NJ, USA) and the CELLQuest software, version 6.0. The platelet population was identified by its forward and side scatter distribution and 20,000 events were analyzed for mean fluorescence.

### Western blot analysis of VASP phosphorylation

Vasodilator-stimulated phosphoprotein (VASP) phosphorylation in washed platelets was additionally determined by Western blot analysis, as previously described^9^. For this purpose, 100 µL of washed platelet suspension was supplemented with 1 mM CaCl_2_ followed by stimulation with buffer, 1 µM DEA/NO or 1 µM PGE1 for 2 min at 37 °C. The cell lysates were loaded onto the gel, separated by SDS-PAGE and then transferred onto nitrocellulose membranes. The membranes were incubated with mouse monoclonal phospho-VASP Ser^239^ (clone 16C2) and phospho-VASP Ser^157^ (clone 5C6) antibodies overnight at 4 °C. For visualisation of the signal, goat anti-mouse IgG conjugated with StarBright Blue 520 was used as secondary antibody, followed by detection with Chemidoc MP imaging system (Bio-Rad Laboratories, Inc., Hercules, CA, USA) and analysis with the corresponding Image Lab Software Version 6.0.

### Platelet aggregation

Light transmission aggregometry was determined in PRP under continuous stirring at 1000 rpm and 37 °C using an APACT 4004 aggregometer (LabiTec, Ahrensburg, Germany). Individual threshold concentrations of agonists (0.5 µM to 3 µM ADP; 0.5 µg/mL to 1 µg/mL collagen) were used to induce submaximal, reversible aggregation. For comparison, irreversible aggregation was determined with 10 µM ADP and 10 µg/mL collagen.

### Adhesion and aggregate/thrombus formation under flow conditions

Adhesion studies and analysis of aggregate/thrombus formation were performed on collagen-coated slides under flow conditions (shear rate 150 s^−1^) at 37 °C using a live cell imaging system with a Nikon Eclipse Ti2 microscope (Nikon GmbH, Duesseldorf, Germany) and a CFI Plan Fluor DLL 60 × oil immersion objective. For this purpose, µ-slides I^0.2^ Luer (ibidi, Graefelfing Germany) were coated with Horm collagen type I (100 µg/mL) for 1 h at RT. Non-adherent collagen was rinsed with HBSS, followed by blocking with modified HBSS buffer [1% (w/v) BSA, 0.1% (w/v) glucose, 0.9% (w/v) NaCl, pH 7.4] for 1 h at RT. For standardization of experimental conditions, platelet count in perfused PRP was adjusted to 3 × 10^8^ platelets/mL using HBSS, supplemented with 1 mM CaCl_2_. Diluted PRP was perfused through the collagen-coated flow chamber for 10 min with the help of a syringe pump at 0.292 mL/min (corresponding to a wall shear rate of 150 s^−1^, simulating arterial shear rates)^27,28^.

The formation of platelet aggregates on the surface of slides was visualized by high-resolution, phase-contrast bright-field imaging (transmitted light microscopy) at six settled spots per sample in an automatic mode. In pre-experiments, the setting of imaging was conditioned to ascertain the analysis of appropriate areas on the slides, where platelets adhere and form aggregates (settled spots). Consecutively, these conditions (in an automated mode, with fixed channel coordinates) were used in the study to image comparable slide areas for all samples. After perfusion, the flow chamber was rinsed with HBSS, and the final image of covered area was used for analysis. The density and the coating thickness of covering platelet layers were determined by analysis of pixel distribution (dependent on the degree of light transmission) and pixel brightness at settled spots. The threshold for “bright pixels” was set at brightness 37,5 (representing approximately 15% of maximal brightness value). For better visualization, black and white was inverted.

### CD62P expression and fibrinogen binding

For determination of basal and TRAP-6-stimulated CD62P expression, 83 µL of PRP were stained with 9 µL of FITC-conjugated anti-CD62P antibody or isotype control for 10 min at 37 °C. After that, 20 µL of platelet suspension were fixed with 1% formaldehyde (final concentration) and the rest was stimulated with 10 µM TRAP-6 for 2, 5 and 10 min at 37 °C. Control and isotype control samples (18 µl PRP + 2 µL FITC-conjugated anti-fibrinogen antibody or isotype control) were stimulated with PBS, simultaneously with TRAP-6-stimulated samples, for 10 min at 37 °C.

For determination of basal and TRAP-6-stimulated fibrinogen binding, 45 µL of PRP were stained with 45 µL of FITC-conjugated anti-fibrinogen antibody for 10 min at 37 °C. After that, 20 µL of platelet suspension were fixed with 1% formaldehyde (final concentration). The residual sample was stimulated with 10 µM TRAP-6 for 2, 5 and 10 min at 37 °C. As control and isotype control served samples (10 µL PRP + 10 µL FITC-conjugated anti-fibrinogen antibody or isotype control for 10 min) stimulated with PBS simultaneously with TRAP-6 samples for 10 min at 37 °C (control).

Finally, platelets were fixed for 10 min at RT, diluted with 300 µL of PBS/BSA/Glc and analyzed by flow cytometry.

### Measurement of chemokine levels

220 µL of WP, prepared from PRP stored at RT, at CT for 1 h or at CT for 2 h, were supplemented with 1 mM CaCl_2_ and stimulated with buffer (control) or 0.5 U/mL thrombin for 30 min at 37 °C. After that, samples were centrifuged for 1 min at 14,000 × *g* and supernatants were divided into 5 tubes and stored at -80 °C. The concentrations of released RANTES, PF4 and sCD40L, were measured by corresponding immunoassay kits.

### Statistical analysis

Descriptive data were calculated with GraphPad PRISM 9 (GraphPad Software, San Diego, CA, USA) and the ImageJ program (National Institutes of Health, USA), similar to previous studies^29^. Data distribution analysis was performed using the Shapiro–Wilk test. Differences of variances between groups were analyzed by one-way analysis of variance (ANOVA) followed by post-hoc Tukey–Kramer-Test for normally distributed data, or by paired Student`s t-test as appropriate. In the case of non-parametric distribution, the Kruskal–Wallis rank based test was used, followed by Dunn’s multiple comparisons test. The comparison of distribution patterns in Fig. 5 was performed with the Kolmogorov–Smirnov test and rank analysis with the two-tailed Mann–Whitney test.* p* < 0.05 was considered statistically significant.

## Supplementary Information

Supplementary Information.

## Data Availability

The datasets generated during and/or analyzed during the current study are available from the corresponding author on reasonable request. All data generated or analyzed during this study are included in this published article.
