# Supplementary figures and images for: The effect of short-term refrigeration on platelet responsiveness
Source: Sci Rep. 2022 Oct 7;12:16910. doi: 10.1038/s41598-022-21124-4 (PMC9546855; doi:10.1038/s41598-022-21124-4)

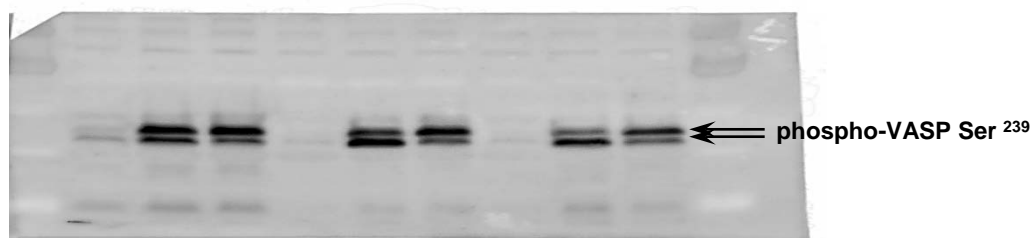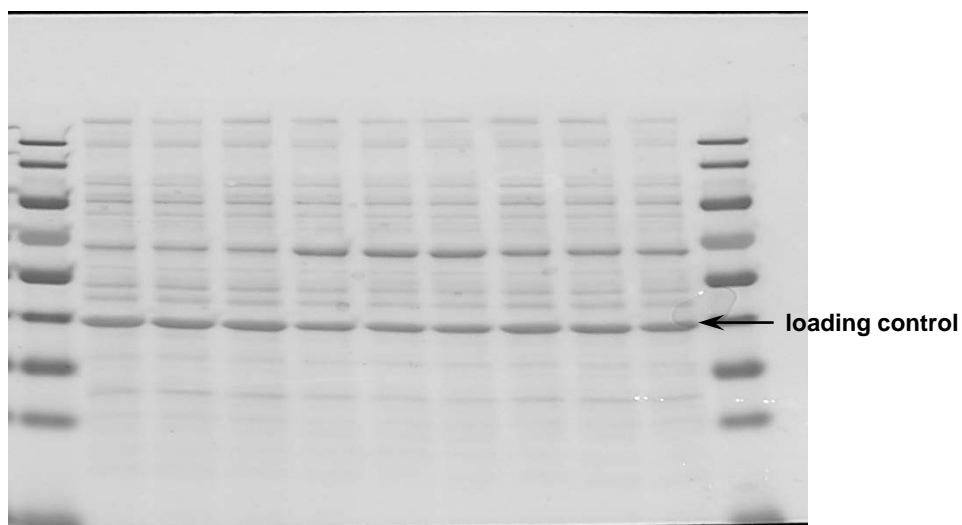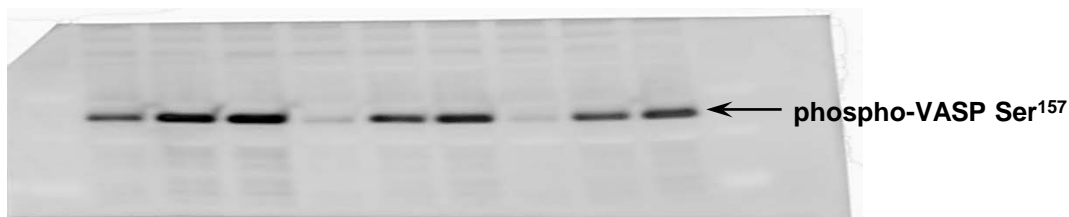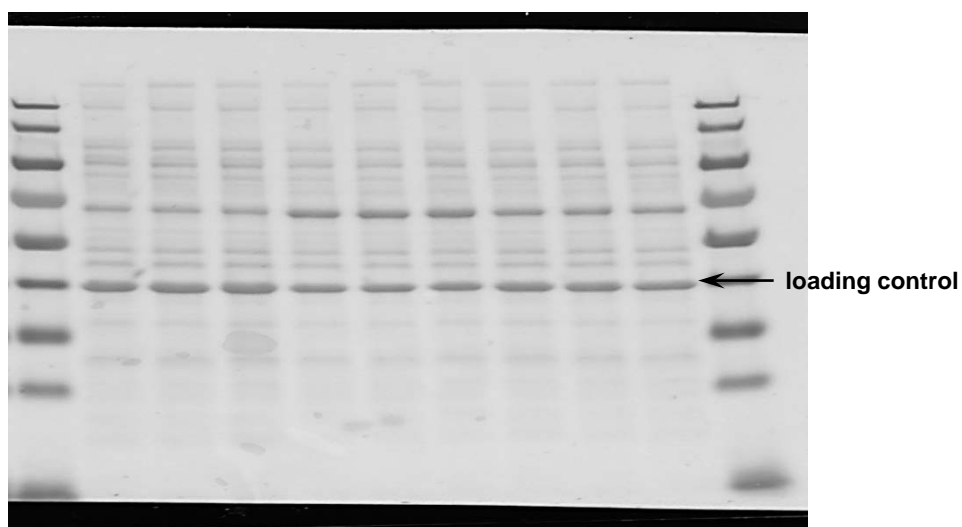

**Figure S1.** The original blots of figure 2a (upper two blots) and figure 2b (lower two blots) are shown.

Supplement: Supplementary file 1 — Supplementary Information. [file 41598_2022_21124_MOESM1_ESM.pdf]
